# Design and Implementation of a Biomimetic Turtle Hydrofoil for an Autonomous Underwater Vehicle

**DOI:** 10.3390/s111211168

**Published:** 2011-11-28

**Authors:** Davinia Font, Marcel Tresanchez, Cedric Siegentahler, Tomàs Pallejà, Mercè Teixidó, Cedric Pradalier, Jordi Palacin

**Affiliations:** 1 Department of Computer Science and Industrial Engineering, University of Lleida, Jaume II, 69, Lleida 25001, Spain; E-Mails: dfont@diei.udl.cat (D.F.); mtresanchez@diei.udl.cat (M.T.); tpalleja@diei.udl.cat (T.P.); mteixido@diei.udl.cat (M.T.); 2 Autonomous System Lab, Tannenstrasse 3 CLA E 14, 8092 Zürich, Switzerland; E-Mails: cedricsi@student.ethz.ch (C.S.); cedric.pradalier@mavt.ethz.ch (C.P.)

**Keywords:** AUV, DoF, sea turtle locomotion, propulsion system

## Abstract

This paper presents the design and implementation of a turtle hydrofoil for an Autonomous Underwater Vehicle (AUV). The final design of the AUV must have navigation performance like a turtle, which has also been the biomimetic inspiration for the design of the hydrofoil and propulsion system. The hydrofoil design is based on a National Advisory Committee for Aeronautics (NACA) 0014 hydrodynamic profile. During the design stage, four different propulsion systems were compared in terms of propulsion path, compactness, sealing and required power. The final implementation is based on a ball-and-socket mechanism because it is very compact and provides three degrees of freedom (DoF) to the hydrofoil with very few restrictions on the propulsion path. The propulsion obtained with the final implementation of the hydrofoil has been empirically evaluated in a water channel comparing different motion strategies. The results obtained have confirmed that the proposed turtle hydrofoil controlled with a mechanism with three DoF generates can be used in the future implementation of the planned AUV.

## 1. Introduction

An Autonomous Underwater Vehicle (AUV) is a robotic device that operates under water and is controlled by an onboard computer. The main advantages of the AUV compared with manned underwater vehicles (MUV) are its reduced size and cost because no human or life-support systems need to be carried, so the operational time depends mainly on battery life. AUVs have many research, commercial, and military applications. One of the earliest AUVs, called Special Purpose Underwater Research Vehicle (SPURV) [1], was developed by the Applied Physics Laboratory at the University of Washington in 1957 to study diffusion, acoustic transmission, and submarine wakes. Currently, the inclusion of specialized sensors in the AUVs has enhanced their ability to perform different applications, such as applied research in biology [2], hydrographic [3], geostatistics [4] or oceanography [5], as well as to detail topological maps of the seafloor [2,6,7] in order to study deep-sea plankton [8], perform mine-countermeasures [3], port protection applications [9], complementing underwater acoustic networks [10,11], and to define the source location of chemical plumes [12].

**Figure 1.**
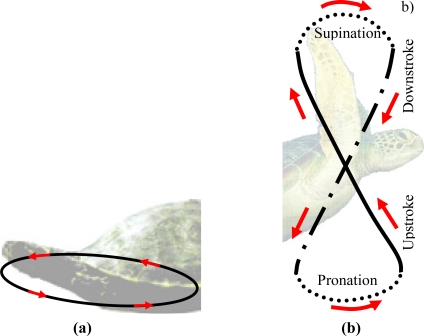
**(a)** Oval path followed by the legs of a freshwater turtle. **(b)** Figure-of-eight path followed by the hydrofoils of a sea turtle.

The most common AUV propulsion system is based on propellers, but they are not a viable solution for applications which require working under low flow conditions, in confined spaces, near the surface [13], and in unsteady flow [14]. The use of fins (hydrofoils) is an alternative propulsion system used by nature, and one which is currently being considered because it allows stable motion with less noise, good flexibility, and high maneuverability [14]. In this work, we propose the design and implementation of a propulsion system for an AUV that is bio-inspired in the turtle’s propulsion (see Figure 1).

### 1.1. Biological Background

The turtle’s anatomy [15] is the biomimetic inspiration for this work. The turtle’s hydrofoil, which is also called fin or forelimb, is its principal source of propulsion. The main characteristic of the skeleton of the turtle’s fin are that the humerus and the radius are very thick but not very long while the phalanx are comparatively very long [15]. These characteristics generate a streamlined hydrofoil that is adapted to swim efficiently because it reduces the resistance of hydrofoil dynamic swirl. In addition, the turtle’s head can also reduce forward resistance by adapting its orientation relative to the navigation path [16].

The turtle’s displacement is produced due to a thrust force generated on both lateral hydrofoils when performing a specific trajectory. Depending on the species of turtle, the trajectory of the hydrofoils changes [17]. Freshwater turtles’ locomotion depends on drag generating thrust by paddling, and each leg follows an oval-shaped trajectory [Figure 1(a)]. The trajectory of the hydrofoil of a sea turtle imitates a trajectory like a figure-of-eight [Figure 1(b)], and both drag and lift forces are involved [18]. In this case, the path followed by the hydrofoils is split into four different phases called downstroke, pronation, upstroke and supination [16,19]. The downstroke and upstroke phases are the most important, whereas pronation and supination are just phases to close the motion cycle with less resistance [18].

### 1.2. Contributions of This Work

This project is part of a larger study under development at the Autonomous System Lab (ASL), department at Eidgenössische Technische Hochschule (ETH), Zürich, where the final target is to develop a turtle-like AUV using hydrofoil propulsion.

To this end, this work proposes the design and implementation of the hydrofoil and the propulsion system for an AUV taking into account that the final fin prototype must be attached in a robot of 1 m length working that must operate in depths up to 10 m. During the evolution of the AUV in the water the assumptions performed were a maximum fluid velocity of 1 m/s and a characteristic hydrofoil linear dimension of 0.1 m, resulting in a constant Reynolds number of 112,359. Based on this result, the hydrodynamic profile selected for the hydrofoil was the National Advisory Committee for Aeronautics (NACA) 0014 because it provided the best relation between lift and drag forces for a constant Reynolds number. Using a similar approach, this profile was used previously in [20] to improve the maneuverability of an AUV.

The selected propulsion path is biomimetically inspired in the sea turtle and will follow a figure-of-eight shape as a way to generate the maximum forward force during the whole period of the motion. The mechanism proposed to generate this hydrofoil displacement is based on a ball-and-socket mechanism with three degrees of freedom (DoF) that is able to replicate any propulsion path for experimentation purposes. Several propulsion paths were tested in a water channel in order to optimize the thrust generated. The proposed propulsion system will be included in a planned future AUV design with turtle-like navigation performances. The external proportions of the different parts of this future AUV will be also bioinspired in the proportions of the sea turtle.

### 1.3. Related Work

The research into AUVs is progressing towards solutions in the commercial, military and research fields. Some examples are Slocum [21,22], based on a buoyancy engine and Ictineu [23], that uses propellers as a propulsion system. In [21] the proposal was the use of the Slocum as a thermal glider using the heat flow between the vehicle engine and the thermal gradient of the ocean temperature in order to propel itself. In this case the control of the pitch and roll was performed by moving an internal mass and the control of the yaw and heading by the hydrodynamic yawing moment due to the roll. In [22] the proposal was the use of an electric glider based on the use of an electromechanical displacement actuator to change their weight. In this case the roll was set by the position of the glider’s static center of gravity (CG) and pitch was controlled by moving its internal mass. Yaw and heading were controlled using the rudder mounted on the vertical tail of the glider. In the case of Ictineu [23], developed by the University of Girona, the AUV prototype was developed to fulfill several aims: moving the robot from a launch/release point and submerging, passing through a 3 × 4 meter validation gate, locating a cross situated on the bottom of the pool and dropping a marker over it, and locating a mid-water target.

Other AUVs, such as Finnegan [14], Madeleine [24], AQUA [25], and NTU turtle robot [26] are examples of robots that alternatively use hydrofoils as a propulsion system to improve maneuverability [20]. Finnegan is a prototype developed by MIT Department of Ocean Engineering Towing Tank, which uses four fins located symmetrically on each side of the robot to generate thrust force. Each fin is started by a pair of actuators allowing an unlimited motion in pitch. The main target of this research was to improve the maneuvering performance of AUVs, while providing the agility to control six degrees of freedom. Madeleine is a prototype developed in 2005 as a result of the cooperation between three institutions: Nekton Research, Monterey Bay Aquarium Research Institute and Vassar College. Like Finnegan, Madeline uses four fins, but in this case, each fin is started by a single actuator. The motivations of this project were to predict efficient fin pitching operation, and build a platform for testing the fin’s locomotion. AQUA is the result of collaboration between McGill and York Universities. This robot is able to swim or walk using six legs, which can be changed depending on the robot’s function. The vehicle uses a variety of sensors to fulfill a range of real tasks in applications that require large autonomy. The NTU turtle robot was developed by Nanyang Technological University. This robot can swim using two fore limbs, which are started by two actuators, while its two hind limbs are used for steering. Finally, a similar approach was proposed in [27] to implement a robotic dolphin.

## 2. Materials and Methods

The materials and methods used to perform the implementation and validation of the proposal can be divided into CAD design, motion and control, water channel, and instrumentation.

### 2.1. CAD Design

Different steps in the work were solved using distinct CAD programs. The JavaFoil platform [28] was used to study the characteristics of the selected hydrodynamic profile to understand how it works and the effects that it generates in the fluid and on the mechanism. The most important information extracted was the estimate of the angle of attack and the lift, drag and momentum coefficients, which allowed the mechanical design to be optimized and the hydrodynamic forces to be calculated. The potential flow analysis performed with this program was a linear varying vorticity distribution. Taking the airfoil coordinates, it was calculated the local, inviscid flow velocity along the surface of the airfoil for any desired angle of attack. First it was calculated the distribution of the velocity on the airfoil surface which could be integrated to get the lift and the moment coefficient. Then it was calculated the behaviour of the flow close to the airfoil surface which was used to calculate the friction drag of the airfoil. Both steps were repeated for the given range of angle of attacks, which yields a complete polar of the airfoil for one fixed Reynolds number. The estimate of the hydrodynamic forces was performed applying a quasi-static analysis at three representative positions of the propulsion system along the sea turtle path and estimating the relative speed at each time.

Working Model (Design Simulation Technologies, Inc., Canton, MI, USA) was used during the design phase in 2D simulations to estimate the torque of the motors. Unigraphics NX 6 (Siemens PLM NX, Plano, TX, USA) and AutoCAD 2010 (Autodesk Inc., San Rafael, CA, USA) were used during the design stage to model the mechanical components. Ansys Workbench 11 (ANSYS, Inc., Canonsburg, PA, USA) was used to simulate each component of the assembly taking into account the maximum equivalent von Mises stress and deformations in order to be sure that all parts were strong enough to withstand the forces while operating in the water channel. Maple (Maplesoft, Waterloo, ON, Canada) and Matlab (Math Works, Natick, MA, USA) were used for calculus, solving system equations and to simulate the displacement of the hydrofoil from the control sequence applied to the motors used to generate the propulsion path.

### 2.2. Motion and Control

In the experimental stage, the mechanical motion of the turtle hydrofoil was generated using Maxon EC 22 50W/167129 (Maxon Motors ag., Switzerland) direct current (DC) motors whose nominal electrical parameters are 32 V, 2.82 A, 50 W, 37.2 mNm, and 20,200 rpm, used in combination with a planetary gearhead GP 32C 190:1. The motor feedback is provided by an optical encoder (MR Encoder 128 CPT) with two channels of 128 counts per revolution and one reference channel.

The control of the absolute angular position of the DC motors was performed by using three EPOS 24/5 from Maxon. The velocity of the DC motors between two angular position points of the defined trajectory was limited to 10,000 rpm. The sequence of angular positions was sequentially defined by using custom control software written in Matlab and C++. The timing and angular sequence applied to the motors was obtained by using a simulation tool written in Matlab that simulates the complete motion of the hydrofoil and the propulsion system in a three-dimensional space depending on the angular position sequence applied to the DC motors.

### 2.3. Water Channel

Experiments were performed at the ETH Zürich in a recirculating water channel 2.5 m long, 0.45 m wide and 0.64 m deep with a 0.4 m water level high. During the experimental phase, the hydrofoil and propulsion mechanism were located at the centre of the water channel whose dimensions were big enough to operate without colliding with the walls.

### 2.4. Instrumentation

The water speed generated on the water channel during the experiments was measured at different points using a helix based MiniWater 6 Micro Schildknecht Anemometer. This device measures water speed from 0.5 to 20 m/s with an accuracy of ±1.0% fs and is unaffected by pressure, temperature, density or humidity. This sensor requires a power supply from 9 to 26 V and its operating range is from −10 to +80 °C for the electronics and from −10 to +140 °C for the probe.

## 3. Design and Implementation of the Turtle’s Hydrofoil

This section discusses the biomimetic design and implementation of the hydrodynamic profile of the turtle hydrofoil.

### 3.1. Hydrodynamic Profile and Optimal Angle of Attack

**Figure 2.**
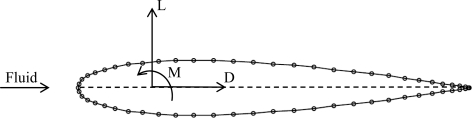
Hydrodynamic forces applied at the pivoting point of a NACA 0014 hydrodynamic profile.

Each hydrodynamic profile in motion in a fluid is influenced by drag *D* and lift *L* forces, and the resulting moment *M* (Figure 2). Lift is defined as the sum of fluid dynamic forces perpendicular to the fluid direction whereas drag is the force that opposes the fluid direction but appears along that direction. All these forces are applied at the pivoting point which is located at 25% of the chord. Equation (1) shows the formulas to compute these forces, which depend on the geometry of the hydrodynamic profile and the characteristics of the fluid involved:

(1) $$\begin{array}{c} L=\frac{1}{2}\cdot V^{2}\cdot \rho \cdot C_{l}\cdot S\\ D=\frac{1}{2}\cdot V^{2}\cdot \rho \cdot C_{d}\cdot S\\ M=\frac{1}{2}\cdot V^{2}\cdot \rho \cdot C_{m}\cdot S\cdot C \end{array}$$

where *V* is the speed of the object relative to the fluid; *ρ* is the density of the fluid; *C* is the chord; *C_l_*, *C_d_* and *C_m_* are the lift, drag and momentum coefficients and *S* is the surface of the hydrofoil. This surface was computed by multiplying the chord *b* by the length of the hydrofoil because the theory of thin profiles can be applied.

The hydrodynamic profile selected for the turtle hydrofoil was the NACA 0014 (Figure 2) because, in simulations with JavaFoil, it provided the best relation between lift and drag forces for a constant Reynolds number (*Re*). This can be computed with:

(2) Re=ρwater⋅V⋅Lμwater

where *ρ_water_* is the density of the fluid; *V* is the fluid velocity; *L* is characteristic linear dimension of the profile and *μ_water_* is the dynamic viscosity of the fluid.

The most important parameter which defines the orientation of the turtle hydrofoil is the angle of attack (α), which is optimal when the lift divided by the drag coefficient is maximum (L/D). In this particular case with a constant speed of the robot of 1 m/s, and 0.1 m as the characteristic linear dimension of the hydrodynamic profile, a constant Reynolds number of 112,359, the optimal angle of attack for the turtle hydrofoil estimated with JavaFoil was 8°. This value will be later verified and optimized experimentally in the water channel.

**Figure 3.**
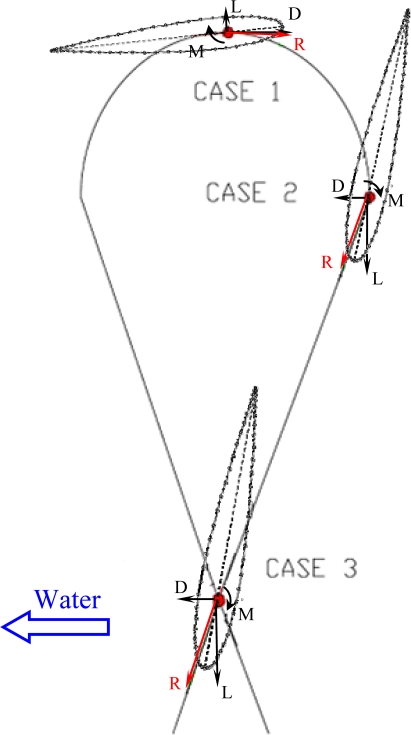
Body diagram of the hydrodynamic profile located at the most unfavorable situations through the eight path (M moment, L lift, D drag, R resultant).

The values of the hydrodynamic forces applied on the static mechanism analysis, deformational analysis and the maximum equivalent stress analysis were computed along the path followed by the sea turtle hydrofoil which has an eight shape. Figure 3 depicts a representation of the trajectory of the turtle’s hydrofoil with three critical positions labelled.

In all cases the profile was titled following the description of the angle of attack. These situations are the most unfavourable since is when the speed of the profile relative to the water is higher and the forces were estimated on the tip of the fin taking into account the hydrodynamic Equations (1). The lift force, drag force and momentum for case 1 are 7.09 N, 0.17 N and −9.3 mNm respectively. For case 2 are 6.88 N, 0.16 N and −9.0 mNm and, for case 3 they are 22.45 N, 0.53 N and −29.3 mNm. Although at high speeds the lift and drag ratios tend to be higher, for this project the Reynolds number was fixed because of the operational specifications planned for the AUV robot.

### 3.2. CAD Design and Implementation

**Figure 4.**
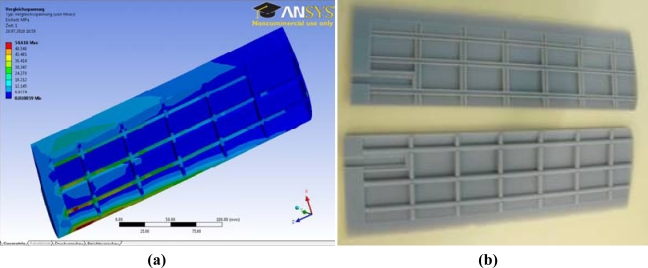
**(a)** Von Mises stress simulation of the internal structure of the turtle hydrofoil. **(b)** Rapid prototype implementation of the turtle hydrofoil.

The design of the turtle hydrofoil was defined by the NACA 0014 profile using a characteristic linear dimension of 100 mm and a profile length of 307 mm, so the aspect ratio of the foil was 3.07. The functional prototype of the hydrofoil will be implemented using a rapid prototype technique in plastic FullCure 720 (tensile strength of 60.3 MPa, tensile modulus equal to 2.87 MPa, shore of 83 and density of 1.094/1.189) by splitting the fin into two parts that will match to become a unique element. The inner part of the hydrofoil was designed sparse to reduce its weight and the Ansys Workbench 11 program was used to verify that the hydrofoil has minimum weight and enough mechanical resistance to operate without a break down in the assembly. The best approach that satisfies both objectives was based on longitudinal strips located at distances of 58, 99.7, 141.4, 183.1, 224.8, 266.5 mm from the button of the fin (part used to attach the fin with the mechanism) and transversal strips located at 9.8, 37.1, 59.3, 71.9 mm from the right part of the fin. The width of the strips was 2.8 mm in both cases. Figure 4(a) shows the results of a von Mises stress simulation of the hydrofoil where the maximum value was 55 MPa, which is lower than the resistance of the material used. Figure 4(b) shows the rapid prototyping implementation of the design. These results confirm that the design will support all the mechanical effort performed without a break down in the assembly.

## 4. Hydrofoil Propulsion Mechanism

In this work, four alternative hydrofoil propulsion mechanisms have been considered: four bar mechanism, differential mechanism, ball-and-socket mechanism, and pulley mechanism. These mechanisms have been analyzed and compared in order to get the best system for the proposed AUV design. The selected propulsion mechanism has been designed and implemented to perform experimental validations in a water channel of the complete hydrofoil propulsion mechanism.

### 4.1. Four Bar Mechanism

**Figure 5.**
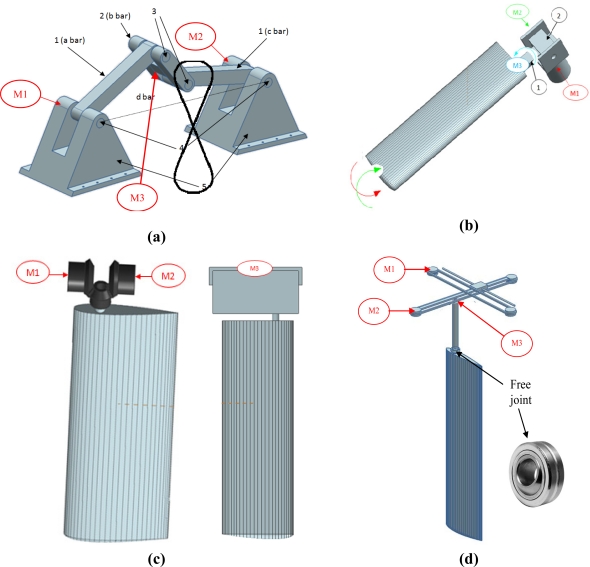
Alternatives considered for the hydrofoil propulsion mechanism: **(a)** 4-bar mechanism. **(b)** Ball-and-socket mechanism. **(c)** Differential mechanism. **(d)** Translational pulley mechanism.

The 4-bar mechanism [Figure 5(a)] has two DoF and consists of four members represented by four bars controlled by two linked motors [M1 & M2: Figure 5(a)] located inside the fixed parts. The hydrofoil is attached to an additional motor placed in the middle point of the *b* bar to change the angle of attack [M3: Figure 5(a)]. The most important characteristic is that the middle point of the *b* bar describes a figure-of-eight trajectory (like the sea turtle). The amplitude of the path depends on the relations between the lengths of the members [Figure 5(a)]. The mechanism is characterized by four singular positions defined when the angle for the input and output bar is 45° and −45° with respect to the *d* bar. In these situations, the mechanism reaches a limit that makes the final motion uncertain. Therefore, in this case, the control of the angular orientation of the two linked motors must work properly to avoid a breakdown in the mechanism.

### 4.2. Ball-and-Socket Mechanism

The ball-and-socket mechanism [Figure 5(b)] has three DoF provided by three motors located strategically in the mechanism and can apply any propulsion path to the turtle hydrofoil. In this case, the horizontal motion is generated by controlling motor M1, motor M2 controls the vertical motion, and M3 is used to control the hydrofoil angle of attack. This design is very compact and can easily be integrated into the structure of an AUV. In this case, the control of the three motors is simple but the generation of the sequence of angular orientations required to generate a figure-of-eight shape trajectory is complex and may require verification with a simulation tool.

### 4.3. Differential Mechanism

The differential mechanism [Figure 5(c)] is a compact mechanism with two DoF currently used for the leg movement in walking robots. In this case, two motors [M1 & M2: Figure 5(c)] feed two bevel gears, located on the horizontal axis, transmitting the motion to a third gear located on a vertical axis. This third gear is able to generate rotation and vertical motions in the hydrofoil. In addition, a third motor [M3: Figure 5(c)] is used to incorporate the third degree of freedom, which corresponds to the horizontal hydrofoil motion. The main drawbacks of this proposal are the large space required inside the AUV and the difficulty of sealing the mechanism properly. In this case, the control of the two linked motors and the generation of the sequence of angular orientation required to control the motors is very simple.

### 4.4. Translational Pulley Mechanism

The pulley mechanism [Figure 5(d)] consists of two pulley transmissions linked by a guide to transmit the axial motion between the two. Each pulley transmission generates motion through a single motor located in a pulley. The combination of two motors [M1 & M2: Figure 5(d)] generates the vertical and horizontal amplitude of the final propulsion path. The hydrofoil is attached to a third motor [M3: Figure 5(d)] to change the angle of attack. The translational movements generated on the transmissions are converted to rotational using a free joint, which is located on the same axis where the hydrofoil is attached. In this case, the main drawback is the flexibility of the ropes that will increase with wear, generating uncertainty in the position of the hydrofoil thus making the mechanical device prone to break downs.

### 4.5. Selected Hydrofoil Propulsion Mechanism

The proposed propulsion mechanisms were compared by simulations using Working Model, Unigraphics NX6, Ansys Workbench and Matlab, considering the relative size and compactness of the different mechanisms, the maximum vertical and horizontal amplitude of the propulsion path achieved, and the maximum torque requirements of the DC motors during the most unfavorable situations along the propulsion path. Table 1 gathers the results of the simulations performed to quantify the selection criteria. Given these requirements, the mechanism selected to propel the hydrofoil was the ball-and-socket mechanism mainly because it is the most compact with a size of 0.01 m^3^ and places few restrictions on the movement of the hydrofoil as it does not have any limitations in the vertical or horizontal amplitude path either. In addition, it was also taken into account the facility of the sealing of the propulsion mechanism inside a real AUV which also agrees with this selection.

### 4.6. Design and Implementation of the Propulsion Mechanism

**Figure 6.**
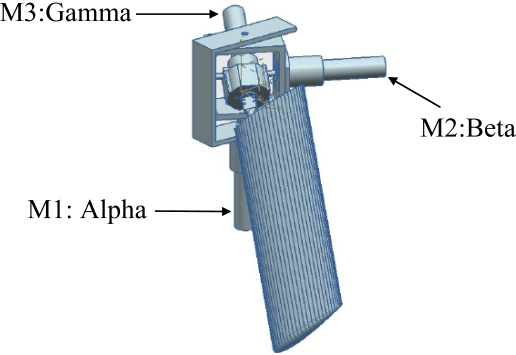
Representation of the hydrofoil and propulsion mechanism. The three motors used are also labeled.

Figure 6 shows the final design of the components involved in the ball-and-socket mechanism with the assembly optimized to work together without collisions. Each component was modeled using Unigraphics NX6 software and the simulations were performed with Ansys Workbench 11. The mesh generated on each component of the assembly was based on Tetra elements using the maximal nodes admitted by the simulator. It was chosen the Tetra elements because it was the ones that took full advantage of object-oriented unstructured meshing technology. The simulation used the CAD geometry components filling the volume with tetrahedral elements using the Octree approach. Once the mesh was created, a static analysis was carried out applying the forces previously estimated at its location. Two additional analyses were performed; a deformational and a maximal equivalent stress analysis. During the analysis different boundary conditions were considered. Some components were studied as a solid rigid (a unique element) because there was not relative motion between them. In addition, in each component analysis a surface was fixed in order to simulate the adequate motion of each part of the assembly in its environment and a pressure force was applied on the surfaces in order to simulate the pressure exerted by the water on the parts. This pressure was calculated taking into account the depth that the robot can reach with:

(3) P=ρ⋅L⋅g

where ρ is the density of the fluid (water = 1,000 Kg/m^3^), L the depth estimate of the robot (in m), and g the constant of gravity (9.81 m/s^2^).

**Figure 7.**
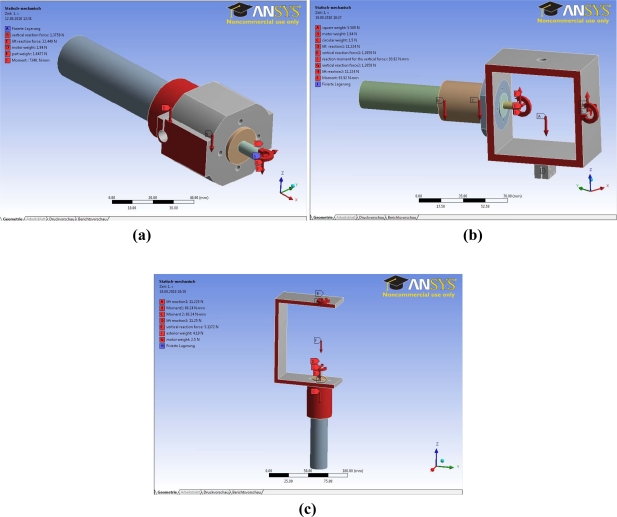
Images of the static mechanical analysis of the hydrofoil propulsion mechanism.

**Figure 8.**
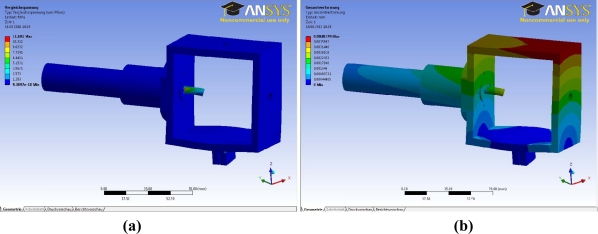
**(a)** Von Mises stress analysis. **(b)** Deformational analysis.

Figures 7 and 8 show the different simulations carried out during the design stage of the ball-and-socket mechanism: Figure 7 shows the static mechanical analysis, Figure 8(a) shows the von Mises stress analysis, and Figure 8(b) the deformational analysis.

Some considerations were made to compute the static mechanical analysis. A rigid solid was created when there was no relative motion between the parts involved, so a simulation study was carried out applying the forces as a unique element. For this, the mechanism was split into three assemblies [Figure 7(a–c)] and the turtle hydrofoil. The first assembly to be studied must be the turtle hydrofoil since is where the most unfavorable hydrodynamic forces were applied and had effects on the other components of the mechanism. Another assumption made was that the hydrodynamic forces were applied to the tip of the hydrofoil.

To ensure that each component will be strong enough to withstand the forces, the maximum von Mises stress results of the simulations have to be higher than the yield stress of the material used to manufacture the part. In addition, the deformations have to be within a limited range to verify that the part will work properly. Figure 8 shows the results of both analyses for one of the assemblies of the mechanism.

**Figure 9.**
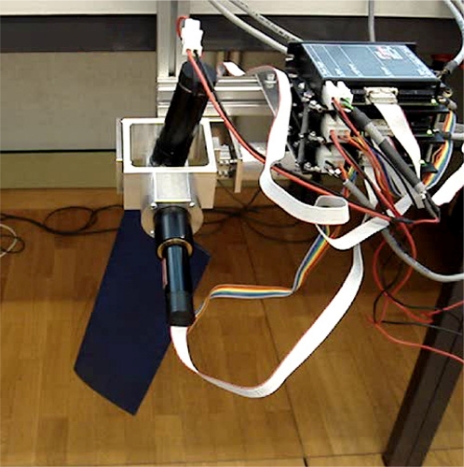
Hydrofoil and mechanical propulsion system.

Finally, Figure 9 shows the practical implementation of the complete hydrofoil and propulsion system. Each motor gives a direct vertical, horizontal and rotational movement to the hydrofoil. The design of the whole mechanism depends on the maximum amplitude of the motion, which was limited in this case to 60° in both directions.

## 5. Experiments

**Figure 10.**
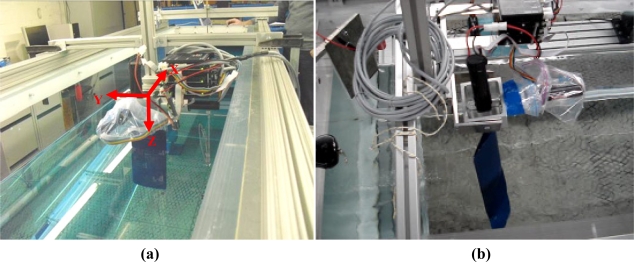
**(a)** Water channel with the reference axis system. **(b)** Water channel facility during an experiment.

In this phase, three experiments were carried out in a water channel to validate the complete design. The propulsion system was fixed in the water channel by a dedicated structure. The main information extracted from the tests was the instantaneous current of each motor during the propulsion path and the average water speed at different points of the water channel. Figure 10(a) shows the reference axis system used in this work and Figure 10(b), an image obtained during the tests.

### 5.1. Experimental Validation of the Optimal Angle of Attack

The first experiment was focused on validating the optimal angle of attack obtained in the design stage. The methodology used in this experiment consisted of applying a predefined fixed propulsion path to the hydrofoil in the water channel and modifying the angle of attack. Each case analyzed was repeated five times and the results were averaged.

**Figure 11.**
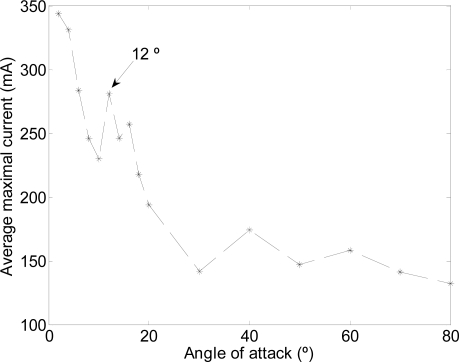
Average maximum current relative to the angle of attack.

The path used was a linear motion where one motor (M1: Figure 6) was activated to generate the amplitude and another motor (M3: Figure 6) was used to define the angle of attack [(a) in Table 2]. Figure 11 depicts the total average maximum current for different angles of attack measured during the linear motion. On one hand, the results show that the total current increases as the angle of attack decreases (increasing the resistance of the water). On the other hand, the maximum water speed in the channel was obtained with an angle of attack of 12°.

The experimental optimal value of the angle of attack was very similar to the value obtained with JavaFoil. The small discrepancy is because the assumptions made during the numerical calculations such as the AUV speed were constant to 1 m/s. In addition, there were some limitations to execute JavaFoil because it does not work with accurate results when working with model laminar separation bubbles and flow separation. Finally, note that this experiment was performed in a water channel where the hydrofoil mechanism was fixed (very far from real AUV operation), so additional empirical validation procedures must be prepared for the final implementation in the AUV.

### 5.2. Experimental Validation of the Optimal Hydrofoil Path

In this experiment, several alternative propulsion paths were compared in order to optimize the AUV’s displacement. Table 2 summarizes the propulsion paths applied to the hydrofoil, the angular position of the motors, the average current of a complete motion, and the maximum current measured. The cases analyzed are (a) linear path with a frequency of 0.36 Hz, (b) diagonal path with a constant angle of attack and a frequency of 0.35 Hz, (c) diagonal path with a variable angle of attack at 0.34 Hz, (d) symmetric figure-of-eight path with a frequency of 0.38 Hz, and (e) anti-symmetric figure-of-eight path with a frequency of 0.38 Hz.

The trajectory in case (a) is a linear motion where an up and down movement is generated while the angle of attack is constant on one way but is changed using the opposite value on the transition between the up and down motion. The absolute value of the angle of attack used was 12°, which was extracted from the experimental tests. The second and third cases reproduce a diagonal motion where the difference was that in case (b) a constant value of the angle of attack of 12° was used whereas in case (c) the angle of attack varied from −94° during the downstroke phase to +56° during the upstroke phase. The value of the angle of attack followed the specification of a sinus wave, which was determined from an analysis of the hydrodynamic forces during a motion cycle in order to optimize the AUV’s displacement. The trajectory of case (d) is a symmetric figure-of-eight path whereas in case (e) an anti-symmetric figure-of-eight path was used. In these two cases, the angle of attack was 12° during the downstroke and upstroke phases but during the pronation and supination phases, this value changed transiently to adopt the correct values on the up and down movement.

During this experiment the propulsion mechanism was fixed to the water channel with a rigid structure while keeping the hydrofoil submerged under the water as much as possible. The motors were covered with a waterproof material to keep them safe. In order to control the propulsion path, the angular orientations of the different motors were sent from one computer to the EPOS. The reverse process was used to extract the position and current information from the motors during the tests. This current was analyzed and averaged in one complete propulsion cycle and the results are shown in Table 2.

The results in Table 2 show that the highest power consumption was obtained in case (c). However, in this case, the thrust generated was not the highest because of some cancelling effects. The cases (d) and (e) had similar propulsion paths and similar average power consumption but case (d) had lower instantaneous current, an aspect that is very interesting in a battery operated AUV. Therefore, according to the results obtained in these experiments, case (d) symmetric figure-of-eight path, is the optimal propulsion path that must be applied to the hydrofoil. This optimal path confirms that the sea turtle is an ideal biomimetic inspiration for the proposed AUV.

### 5.3. Water Speed Measurements

**Figure 12.**
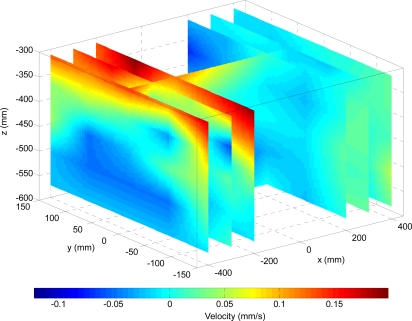
Four-dimensional graph of the evolution of the water speed along the water channel while running the optimal propulsion path.

The last experiment consisted of the measuring the channel water speed (Figure 12) while applying the proposed optimal propulsion path to the hydrofoil (symmetric figure-of-eight path). The measurements points are specified relative to the (0,0,0) position which is defined at the center of the propulsion mechanism [see Figure 9(a)], the water level was at −200 mm in the z axis to simplify sealing the complete mechanism. The water speed was measured at the intersection of the mesh defined by the planes: [−400, −300, −200, 200, 300, 400] mm along the x axis, [−150, −75, 0, 75, 150] mm along the y axis, and [303.7, 370.4, 437.1, 503.8, 570.5] mm along the z axis [reference axes in Figure 9(a)]. To perform the measurements, the anemometer was introduced into the water channel together with a rigid structure to prevent the ripples created in the water by the force generated by the hydrofoil during motion from moving the sensor.

Figure 12 shows the measurement of the water speed generated in the water channel while applying the optimal propulsion path to the hydrofoil. Figure 12 depicts a four-dimensional graph that represents the water speed profile interpolated from the measurements taken. In general, the water speed is higher closer to the surface probably because the propulsion mechanism is not completely submerged. In this case, the water speed is higher for negative X coordinates (−400, −300, −200 mm) whereas on positive X coordinates (200, 300, 400 mm), it is lower because the movement of the hydrofoil has to propel the AUV on that direction. Figure 12 also shows the water speed profile in different YZ sections. The speed on these surfaces decreased from the upper to the bottom even being negative in some zones as a consequence of vortices. In addition, on the three sections located on positive X coordinates, the overall speed on the entire surface was higher on X negative coordinates (immediately backwards the hydrofoil), which is consistent with the results achieved for the XZ section.

## 4. Conclusions

This work presents the biomimetic design and implementation of a hydrofoil propulsion system for a turtle-like Autonomous Underwater Vehicle (AUV). The hydrofoil design was based on a NACA 0014 hydrodynamic profile that is functionally very similar to a sea turtle’s fin. The proposed design of the hydrofoil was analyzed to reduce weight and ensure resistance. Four propulsion mechanisms were proposed and evaluated in terms of compactness, motion amplitude, sealing, and torque requirements, and a ball-and-socket mechanism was selected to propel the hydrofoil, mainly because it is very compact and has very few movement restrictions. The proposed propulsion mechanism was also analyzed to reduce weight and ensure resistance. Finally, theoretical and simulation results have been validated experimentally. A first experiment was performed to validate the optimal angle of attack of the hydrofoil. A second experiment was performed to validate the optimal propulsion path of the hydrofoil. The best results were obtained when using the sea turtle’s path. A third experiment was performed to measure the profile of the water speed generated by the displacement of the hydrofoil. The highest water speed was obtained on the surface of the water channel, probably owing to the mechanical propulsion system not being completely submerged. Therefore, the conclusion of this work is that the design and implementation of an AUV can be optimized and enhanced when using nature for biomimetic inspiration. Specifically, in this work, the sea turtle inspired the design of the hydrofoil and the propulsion path for an AUV. Future work will involve the design of a whole turtle-like Autonomous Underwater Vehicle.

## Figures and Tables

**Table 1.** Decision matrix.

**Factors**	**Alternatives**			
	**4-bar mechanism**	**Differential mechanism**	**Ball and socket mechanism**	**Pulleys (free point)**
**Size**	0.0108 m^3^	0.074 m^3^	0.01 m^3^	0.012 m^3^
**Maximum vertical amplitude path**	0.32 m	No limit	No limit	0.346 m
**Maximum horizontal amplitude path**	0.2 m	No limit	No limit	0.346 m
**Torque (M1,M2,M3)**	0.0226 Nm 0.0226 Nm 0.0075 Nm	0.0226 Nm 0.0226 Nm 0.0677 Nm	0.0015 Nm 0.0601 Nm 0.0601 Nm	0.0526 Nm 0.0526 Nm 0.075 Nm

**Table 2.** Propulsion paths.

		**Description of the motion**	**Motor position (M1, M2, M3)**	**Mean current (mA)**	**Maximum current (mA)**
**(a)**	linear path	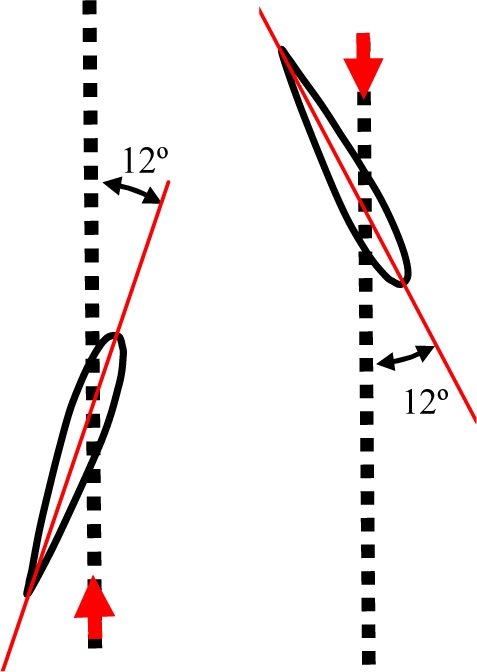	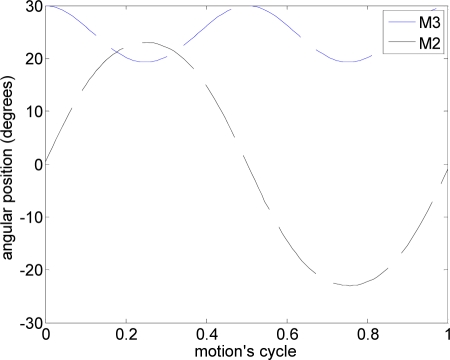	103.0	518
**(b)**	diagonal path with a constant α	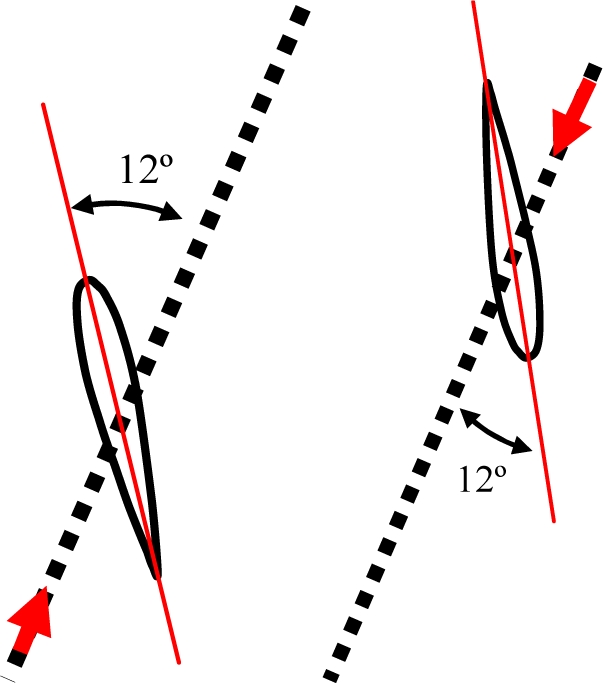	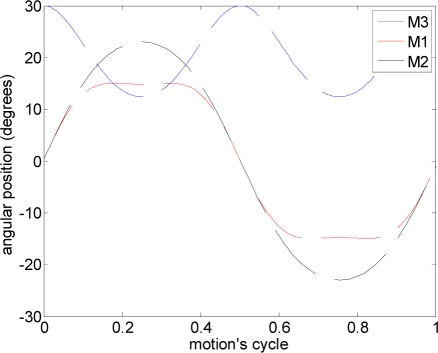	115.5	515
**(c)**	diagonal path with a variable α	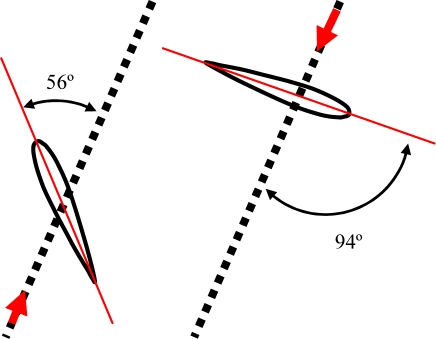	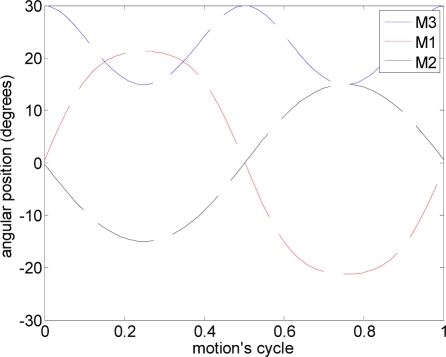	144.2	601
**(d)**	symmetric figure-of-eight path	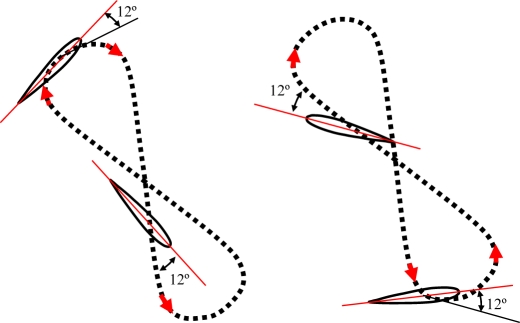	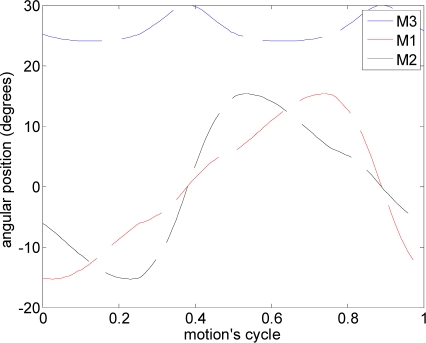	129.4	657
**(e)**	anti-symmetric figure-of-eight path	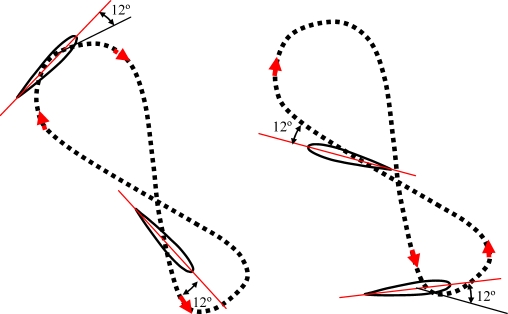	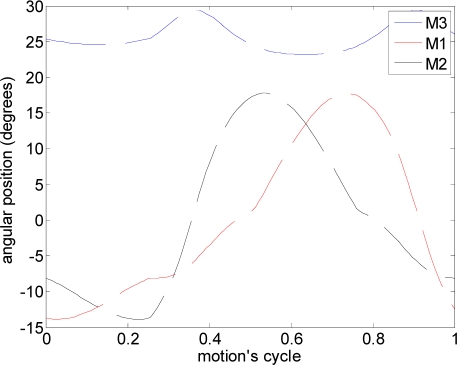	130.0	851
